# Multifunctional Sodium Hyaluronate/Chitosan Foam Used as an Absorbable Hemostatic Material

**DOI:** 10.3390/bioengineering10070868

**Published:** 2023-07-21

**Authors:** Ran Chen, Fanglin Du, Qipeng Yuan

**Affiliations:** 1 Laboratory of Biosynthesis and Efficient Separation of Natural Active Ingrediens, Beijing University of Chemical Technology, Beijing 100029, China; 2 State Key Laboratory of Organic-Inorganic Composites, Beijing Laboratory of Biomedical Materials, Beijing University of Chemical Technology, Beijing 100029, China

**Keywords:** hyaluronic acid, carboxymethyl chitosan, composite material, hemostatic foam, biodegradable

## Abstract

Absorbable hemostatic materials have great potential in clinical hemostasis. However, their single coagulation mechanism, long degradation cycles, and limited functionality mean that they have restricted applications. Here, we prepared a sodium hyaluronate/carboxymethyl chitosan absorbable hemostatic foam (SHCF) by combining high-molecular-weight polysaccharide sodium hyaluronate with carboxymethyl chitosan via hydrogen bonding. SHCFs have rapid liquid absorption performance and can enrich blood cells. They transform into a gel when it they come into contact with blood, and are more easily degraded in this state. Meanwhile, SHCFs have multiple coagulation effects and promote hemostasis. In a rabbit liver bleeding model, SHCFs reduced the hemostatic time by 85% and blood loss by 80%. In three severe and complex bleeding models of porcine liver injury, uterine wall injury, and bone injury, bleeding was well-controlled and anti-tissue adhesion effects were observed. In addition, degradation metabolism studies show that SHCFs are 93% degraded within one day and almost completely metabolized within three weeks. The absorbable hemostatic foam developed in this study is multifunctional; with rapid hemostasis, anti-adhesion, and rapid degradation properties, it has great clinical potential for in vivo hemostasis.

## 1. Introduction

Uncontrolled bleeding can lead to complications such as coagulation dysfunction, acidosis, and organ failure, greatly increasing patient mortality rates [1,2,3,4]. In these circumstances, emergency and effective hemostatic measures are crucial for saving the patient’s life. Various hemostatic materials have been developed [5,6,7,8,9,10,11,12]; for instance, inorganic clays such as zeolite and kaolin are effective methods for managing traumatic bleeding [13,14,15]. Various composite dressings are widely used, such as QuikClot Combat Gauze™ (QCG), approved by the FDA in 2013. This dressing is made by impregnating gauze with kaolin, and has a rapid hemostatic effect. Chan et al. [16] modified a chitosan dressing with PolySTAT, a synthetic polymer that enhances coagulation via cross-linking fibrin, improving its hemostatic performance. However, materials such as inorganic clays may carry safety risks, including exothermic reactions and thrombosis, and most existing hemostatic materials are non-degradable and suitable only for external traumatic bleeding [17,18]. Non-absorbable materials that remain in the body for a long time may cause wound inflammation, hinder wound healing, and lead to other complications, and their removal via surgery increases the risk of infection as well as the cost of treatment [19,20].

Absorbable hemostatic materials are widely researched due to their biodegradability, metabolism in the body, and lack of need for removal [21,22,23]. Natural polysaccharides such as cellulose, chitosan, and starch are widely used in hemostatic materials, especially the absorbable kind, due to their excellent biocompatibility and biodegradability [10,24,25]. Structural proteins such as collagen and gelatin are used in the development of absorbable hemostatic materials as well. However, existing absorbable materials have a relatively simple mechanism of action and are not effective enough to treat complex wounds such as gynecological and orthopedic bleeding [22,26,27]. Moreover, they have a long degradation cycle in the body; for example, regenerated oxidized cellulose takes 4–8 weeks to completely metabolize [28,29]. Abnormal adhesion between postoperative organs or tissues can cause pain, discomfort, and functional impairment. Absorbable hemostatic materials should have anti-adhesion properties [30,31,32,33].

Chitosan is a representative polysaccharide material with excellent properties, including biocompatibility, mucosal adhesion, and the cationic enrichment of blood cells, making it suitable for hemostasis [21]. However, hydrogen bonding interactions between and within chitosan molecules cause poor water solubility and weaken hemostatic effects [34,35]. Carboxymethyl chitosan (CMCH) can be obtained by modifying chitosan, which improves its water solubility [36,37]. High-molecular-weight sodium hyaluronate (SH) has good water absorption and gelation properties, and can act as a good barrier between tissues and organs, reducing friction between tissues and minimizing adhesions [38,39,40,41,42]. Composite strategies are usually used to combine the characteristics of two or more materials, addressing the shortcomings of single hemostatic materials and achieving multifunctionality [43,44,45,46]. Guo et al. [47] prepared a series of dry cryogel hemostatic agents by combining gelatin and dopamine, simultaneously achieving hemostasis of deep non-compressible wounds and improving wound healing.

To achieve rapid hemostasis, anti-adhesion, and rapid degradation in a multifunctional composite, we combined two high-molecular-weight polysaccharides with excellent biocompatibility and biodegradability using hydrogen bonding interactions to prepare an absorbable hemostatic foam (SHCF). Briefly, sodium hyaluronate and carboxymethyl chitosan powder were weighed, dissolved in deionized water, and continuously stirred at room temperature. They were then allowed to swell until completely dissolved. The two solutions were mixed, stirred well, and then loaded into molds. They were left to dry to obtain a hemostatic foam.

SHCFs can rapidly absorb liquid and enrich blood cells. Due to its non-covalent binding mode an SHCF can form a water gel that adapts to and seals a wound after contact with blood; this makes it more easily degradable as well. Moreover, SHCF exhibits strong coagulation stimulation, promoting hemostasis. The hemostatic performance of the SHCF was evaluated using a rabbit liver bleeding model. The hemostatic and anti-adhesion properties of the SHCF for complex and severe bleeding wounds were evaluated using three bleeding models in porcine animals: liver injury, uterine wall injury, and bone injury. In addition, the absorbability of the SHCF was evaluated using in vitro degradation, in vivo metabolism, and subcutaneous implantation tests.

## 2. Materials and Methods

### 2.1. Materials

Sodium hyaluronate (SH), MW = 1,400,000–2,000,000 g/mol, was purchased from Bloomage Biotechnology Co., Ltd., Shangdong, China. Carboxymethyl chitosan (CMCH), MW = 10,000–20,000 g/mol, degree = 91.8% concentration, deacetylation degree = 80%, was purchased from Bloomage Biotechnology Co., Ltd., Shangdong, China. Chitosan-based absorbable hemostatic material (SURCHI) and hyaluronic acid-based absorbable hemostatic material (AHF) were purchased from Qingdao Boyit Biomaterials Company (Qingdao, China).

### 2.2. Preparation of Sodium Hyaluronate/Carboxymethyl Chitosan Absorbable Hemostatic Foam (SHCF)

Sodium hyaluronate powder was accurately weighed, slowly added to deionized water, and continuously stirred for 2 h at room temperature with an electric mixer at a rate of 300 ± 20 r/min. It was then allowed to swell for 2 h until completely dissolved, reaching a final concentration of 0.2~0.4% (w%). Carboxymethyl chitosan was dissolved using the same method, with a final concentration of 0.1~0.2% (w%). After the complete dissolution of carboxymethyl chitosan, viruses such as vesicular, non-vesicular, DNA, and RNA viruses were inactivated via high temperature treatment (100 °C, 15 min). The above two solutions were mixed in a 1:1 mass ratio and stirred until the mixture was homogeneous. The homogeneously mixed solution was loaded into the mold for drying to obtain a hemostatic foam. The thickness of the obtained foam was approximately 5 mm.

### 2.3. Characterization of SHCF

Scanning electron microscopy (Gemini SEM 300, voltage 3.00 kV; the sample was sprayed with gold for 30 s) was used to observe the SHCF structure. SEM samples were prepared by cutting and gluing samples onto conductive adhesives, then spraying with gold for 30 s using a Quorum SC7620 radio coater (spraying current of 10 mA). Fourier-transform infrared spectroscopy (FT-IR, THERMO FISHER Nicdet IS5, number of scans = 32) was used to test the SH, CMCH, and SHCF in the wave number range of 4000–500 cm^−1^. In a dry environment, the foam was ground into a powder and mixed with potassium bromide at 1:50 (w%). The mixed powder was then pressed into thin sheets. Energy-dispersive spectroscopy (EDS, Hitachi S-4700, low beam energy = 12 kV, working distance 10–14 mm) was used to analyze the elemental content of SHCF. XPS analysis was run on a Thermo Scientific K-Alpha using a monochromatic Al Ka source with an energy of 1486.6 eV and working power of 150 W. Peaks were analyzed using the nonlinear fitting software XPSPEAK 4.1. The Brunauer–Emmett–Tellerm (BET) method was carried out using an Autosorb-iQ-MP automated physisorption instrument (USA). A dried sample of about 80 mg was dehydrated in vacuum at 200 °C for 8 h, then the N_2_ adsorption and desorption curves were performed at a liquid nitrogen temperature of −198 °C. The relative pressure range of the adsorption point was 10^−2~1^ (*p*/p_0_), the nitrogen adsorption amount at the pressure point (*p*/p_0_ = 0.99) was taken as the total pore volume, and the specific surface area of the samples was calculated using the BET method. The samples were cut into strips of 10 mm width and then loaded into a universal testing machine INSTRON 5982 (USA) to test the elastic modulus. The loading spacing was 20 mm and stretching was performed at a speed of 10 mm/min until the sample was broken.

### 2.4. Water Absorption Performance Test

Contact angle meters (CA100A) were used to determine the water absorption rate of the SHCF, SURCHI, and AHF. The surface-free energy of the SHCF was analyzed in contact mode using an AFM A-100 atomic force microscope (APE Research-Italy) under ambient conditions. The AFM tip had a diameter of approximately 8 nm, a cantilever of 450 ± 5 μm in length, a resonance frequency of 13 kHz, and a sampling area of 4 μm × 4 μm. The scan rate and length were 2000 nms^−1^ and 1 μm. Equal masses of SHCF, SURCHI, and AHF were weighed and immersed in deionized water for 5 min. The respective weight of the material after water absorption was recorded and the water absorption multiplier was calculated using the following formula: Abs. weight = (W_2_ − W_1_)/W_1_, where W_1_ and W_2_ respectively represent the weight before and after the SHCF absorbed water.

### 2.5. Gel-Forming Performance Test

Six pieces of SURCHI, six pieces of medical petroleum jelly gauze, and six pieces of SHCF, all 5 cm × 5 cm in size, were collected. Next, 10 mL of anticoagulated rabbit blood was added to completely infiltrate the foam; the samples were observed to be gel-forming within 5 min. MCR 302 (Anton Paar, Graz, Austria) was used to test the rheological behavior of SHCF gel. The sample was cut into 25 mm diameter discs and loaded in the middle of the test bench. The measuring rotor was moved downward to ensure that the rotor touched the sample. The test began in the range of 0.1–100 Hz.

### 2.6. In Vitro Dynamic Coagulation

SHCF samples were cut into 1 cm × 1 cm segments and placed in the center of a Petri dish, then 100 μL of anticoagulated blood was added to the surface of the samples, followed by the immediate addition of 10 μL of 0.2 M CaCl_2_ solution used as a coagulant. Every 5 min, 20 mL of deionized water was slowly added to one of the Petri dishes along the wall, taking care to avoid damage to the already clotted blood. The absorbance of the post-hemolysis solution was then measured at 545 nm using a UV spectrophotometer. At the same time, 20 mL of deionized water was added to 100 μL of blood to detect its absorbance as the absorbance value at point 0. Three parallels were made for each sample.

### 2.7. In Vitro Red Blood Cell (RBC) Adsorption

Anticoagulated whole blood was centrifuged at 1500 r/min for 15 min to produce a 2% RBC suspension. Take 100 μL of the erythrocyte suspension and drop it onto the surface of the 1 cm × 1 cm material. Every 5 min, 20 mL of deionized water was slowly added to the Petri dish along the wall, taking care to avoid damaging the already coagulated erythrocytes. The absorbance of the post-hemolysis solution was then measured at 545 nm using a UV spectrophotometer. At the same time, 20 mL of deionized water was added to 100 μL of red blood cell suspension and its absorbance was measured as the absorbance value at point 0. The red blood cells that were not adsorbed around the material were rinsed off with saline and then placed in a drying oven at 37 °C for 30 min. The surface erythrocyte morphology was observed by scanning electron microscopy using gold spray under vacuum conditions.

### 2.8. In Vivo Hemostasis and Anti-Adhesion Evaluation

All animal experiments were conducted in accordance with the protocol approved by the animal experimental center of Nongnong Life Science & Technology Company, Beijing, China (no. 20200302-115, 20200302-116, 20200302-117, 20200302-118).

For the rabbit liver injury hemorrhage model, New Zealand rabbits (2.8–3.2 kg) were anesthetized via intraperitoneal injection of 10% chloral hydrate (1 mL per 200 g rabbit weight). The thoracic cavity was opened with surgical scissors and wounds were made in the liver using a scalpel (2.5 mm wide). Immediately afterwards, the bleeding was stopped by applying SHCF to the wound. The bleeding time and amount of blood loss were recorded after the end of the procedure. The weight and body temperature of the animals were recorded for 14 days after the experiment.

For the porcine liver injury hemorrhage model, Guizhou small porcines (18–35 kg) were anesthetized, the liver was openly exposed, and a 2.5 mm wide wound was constructed in the middle part of the liver lobe. Hemostasis was performed using an SHCF cover over the wound, then the times of hemostasis and blood loss were recorded. After dissection, wounds were observed for adhesion production and graded according to the Phillips five-level method.

For the porcine uterine wall hemorrhage model, after the animals were anesthetized the uterus was exposed by opening the abdomen, a 2 cm long wound was created in the uterine wall to a depth of the myometrium to the plasma layer of the uterine wall, two or three injuries were made per animal, sutures were applied, and the material was used. Hemostasis was performed and evaluated by exuding blood from the wound, then the times of hemostasis and blood loss were recorded. In post-dissection animals, the wounds were observed for adhesion production and graded according to the Phillips five-level method.

For the porcine bone injury hemorrhage model, after animals were anesthetized, a 5 cm long wound was created in the hind limb to expose the tibia and an injury of approximately 5 mm in diameter and 5 mm in depth was created in the tibia, reaching the cancellous bone uncovered to the bone marrow cavity. Two or three defects were created in each tibia for hemostatic evaluation, then the hemostatic time and blood loss were recorded.

### 2.9. Evaluation of Cytotoxicity and Hemolysis Rate

The experiments were performed following the International Standard ISO 10993 (Biological evaluation of medical devices). The number of L929 cells (from Cell Resource Center, IBMS, CAMS/PUMC, Beijing, China) was adjusted to 8 × 10^4^ cells mL^−1^ in complete medium (CM), which consisted of 90% RPMI-1640 medium, 10% fetal bovine serum (FBS), and 1% antibiotics (100 units mL^−1^ penicillin and 100 units mL^−1^ streptomycin). The cell suspensions were added to 96-well plates (0.1 mL for each well) for 24 h at 37 °C (5% CO_2_). Then, the extraction solutions (RPMI 1640 medium was used at an extraction ratio of 1.25 cm^2^/mL for 24 h at 37 °C) were added to 96-well plates after the original medium was removed. The plates were cultivated for 24 h and PBS was subsequently employed to wash cells twice. Measurements were performed at 570 nm using an enzyme marker at the end of the incubation. The blank control group was recorded as 100%; for each other dose group and control group, the relative cell proliferation rate was calculated by comparing the OD value to that of the blank control group.

Next, 200 µL of RBC suspension was mixed with 800 µL of SHCF solution (3 mg/mL) and placed in an oven at 37 °C for at least 3 h, during which the tubes were turned upside down every 30 min to ensure that the material was in full contact with the red blood cells. The absorbance was then measured at 540 nm and the hemolysis rate was calculated using the following formula: hemolysis (%) = (Abs_sample_ − Abs_negative_)/(Abs_positive_ − Abs_negative_) × 100%, where Abs_sample_, Abs_negative_, and Abs_positive_ are the absorbance values of the sample group, the negative group (PBS), and the positive group (water) at 540 nm, respectively.

### 2.10. Inflammatory Factors Level Test

In the experimental group, samples were subcutaneously implanted on the back of mice; in the negative control group, the same treatment was used with no samples implanted. In the positive control group, 20 mg of BSA was mixed with 10 mL of PBS (pH 7.4) and then mixed with an equal volume of Freund’s adjuvant to form an emulsion, then 0.10 mL was subcutaneously injected in the back of each animal once every 10 days for a total of three times. Blood was collected from the mice via orbital blood collection; the blood was stored overnight at 4 °C and centrifuged at 1500× *g* for 10 min, then the supernatant was collected. The serum was assayed for IL-1β, IL-6, and TNF-α using commercially available ELISA kits according to the instructions.

### 2.11. In Vitro Degradation Test

For enzyme solution preparation, enzyme solution was prepared using sterilized PBS. The concentration of hyaluronidase in the solution was 0.25 mg/mL and the concentration of lysozyme was 0.023 mg/mL. The solution was stored at 4 °C. SHCF 0.88 g was weighed and added to sterilized PBS (40 mL) and enzyme solution (40 mL), respectively, to prepare a solution with a sample concentration of 22 mg/mL. Each group was equally divided into four portions: three for the viscosity assay and one for the molecular weight assay. At the same time, 0.88 g of control product was weighed and 40 mL of enzyme solution was added to prepare a solution with a concentration of 22 mg/mL for the molecular weight assay. All of the above solutions were incubated at 37 °C.

For the viscosity assay, the kinetic viscosity and characteristic viscosity numbers of PBS and enzyme degradation were monitored at 0, 1, 3, 6, 12, 21, and 28 days. Kinetic viscosity was measured using a rotational viscometer at room temperature. The characteristic viscosities were measured using a Ubbelohde viscometer, which was placed in a water bath at 25 °C to ensure a stable temperature in the testing room. The SHCF was dissolved in PBS and enzyme solution in g/dL and placed in the viscometer. The efflux times of the solutions were measured using the viscometer, and the pure solvents were similarly assessed. The intrinsic viscosity number was calculated using the formula [η] = lnηr/c, where ηr is T/T_0_, T_0_ is the time taken for the level of pure solvent to decrease, T is the time taken for the SHCF solution to decrease, and c is the concentration of the SHCF solution.

For the molecular weight assay, 5 mL of enzyme solution was collected at 0, 1, 3, 6, 12, 21, and 28 days and the molecular weight before and after degradation was measured using GPC gel chromatography.

### 2.12. Metabolic Studies

For Solution 1, 630 µCi of [3H] sodium hyaluronate was mixed with 129.2 mg of carboxymethyl chitosan. For Solution 2, 630 µCi of [3H] carboxymethyl chitosan was mixed with 129.2 mg of carboxymethyl chitosan. Rats were intraperitoneally injected with 20 mL/kg of both solutions, then their body weight and actual dosing amounts were recorded. The rats were quickly transferred back to the experimental cage apparatus for collection of urine and fecal samples. The samples were then added to a scintillation solution to determine their radioactivity.

### 2.13. Subcutaneous Implantation Test

Sodium pentobarbital was intravenously administered at a dose of 40 mg/kg to anesthetize Balb/c mice. The dorsal hair of the test animals was removed. Subcutaneous implantation was performed along the left and right sides of the median line of the spine of the mice. At 4, 12, and 16 weeks of implantation, the skin at the implantation site was marked with picric acid; at 20 and 24 weeks of implantation, a small section of surgical thread was added to mark the implantation site of the samples. The tissues were subjected to HE staining for observation.

## 3. Results and Discussion

### 3.1. Synthesis and Characterization of SHCF

**Figure 1:**
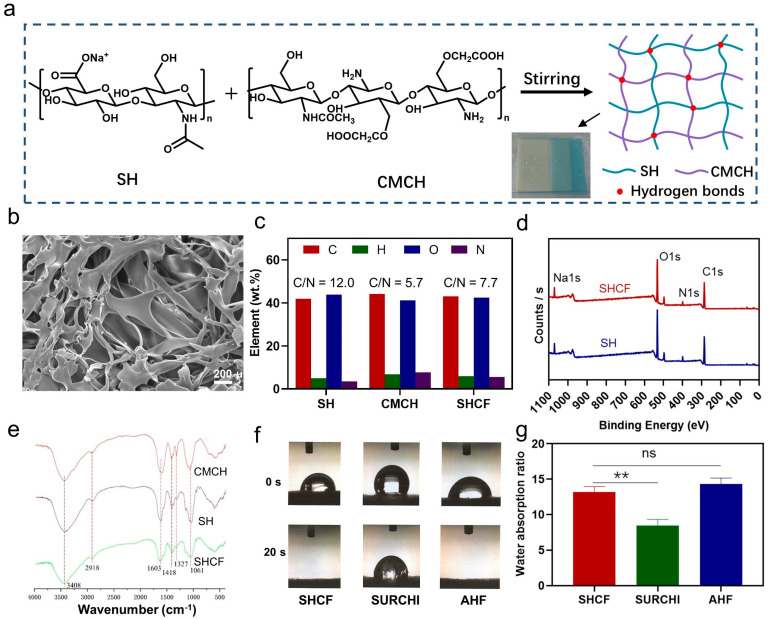
(**a**) Route of SHCF synthesis; (**b**) SEM photographs of resulting SHCF; (**c**) elemental analysis; (**d**) XPS analysis; (**e**) FT-IR results for SH, CMCH, and SHCF; (**f**) liquid absorption time; (**g**) absorption weight of SHCF, SURCHI, and AHF. Error bar indicates SD (n = 3). ** *p* < 0.01.

We synthesized a sodium hyaluronate/carboxymethyl chitosan absorbable hemostatic foam (SHCF) through the formation of hydrogen bonding interactions (Figure 1a). SEM images show that the SHCF has abundant internal pores and that these pores are interconnected (Figure 1b). BET analysis shows that the surface area and pore volume of SHCF are 6.974 m^2^/g and 0.007 cm^3^/g (Table S1), respectively, which are high values, suggesting that the SHCF has a rich porous structure. Elemental analysis results (Figure 1c) show that the C/N ratios of SH and CMCH are 12.0 and 5.7, respectively. The C/N ratio of the SHCF obtained from the combination of the two is 7.7, which corresponds to the reactant ratio (SH: CMCH = 1:1). XPS analysis (Figure 1d) shows that the peaks of Na, O, N, and C appear in both the SH and SHCF spectra and that the ratios of each element correspond to the ratios of the reactants. These results confirm the successful synthesis of the hemostatic foam. SH, CMCH, and SHCF all show asymmetric stretching vibration absorption peaks, while symmetric stretching vibration absorption peaks of carboxyl groups appear at 1603 cm^−1^ and 1418 cm^−1^ in the FT-IR spectra. The absorption peak at 1601 cm^−1^ has a stretching vibration peak of C-O. Therefore, the characteristic peaks of the SHCF are in the same positions as those of the SH and CMCH, with no newly emerging characteristic peaks (Figure 1e). These results indicate that the two polysaccharides were physically mixed through hydrogen bonding interactions rather than chemically reacting to produce new covalent bonds. Overall, these results demonstrate successful SHCF synthesis.

### 3.2. Liquid Absorption and Gel-Forming Properties of SHCF

Hemostatic materials should have the ability to quickly absorb liquids. The liquid absorption ability of the SHCF was evaluated and compared to two commercial absorbable hemostatic materials, SURCHI and AHF. The SHCF quickly absorbed water within 20 s, which was comparable to the absorption rate of AHF. The contact angle was 72.01 ± 0.91° and the surface free energy of the SHCF was 37.30 ± 1.4 mJ·m^−2^, which corresponds to the contact angle data (Figure S1 and Table S3). Therefore, the SHCF exhibits good liquid absorption performance. In contrast, SURCHI could not completely absorb water in the same time period (Figure 1f). The SHCF was able to absorb water up to 13.16 times its own weight, which was significantly more effective than the control group (SURCHI). This demonstrates the fast liquid absorption ability of this type of foam (Figure 1g) due to the excellent hydrophilicity of SH and CMCH and the rich porous structure inside the SHCF. These results indicate that SHCFs can be beneficial for quickly absorbing water in the blood and enriching blood cells, thereby promoting blood clotting.

**Figure 2:**
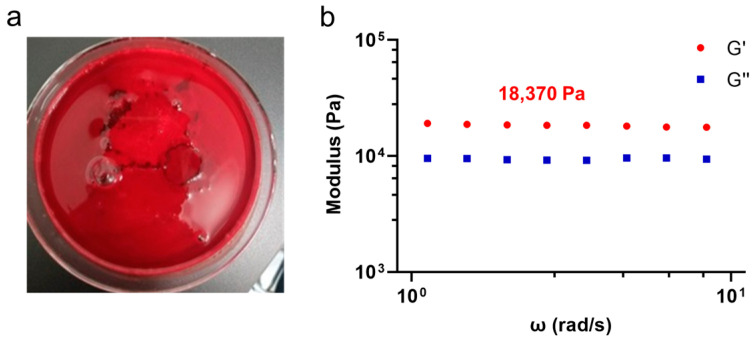
(**a**) Photograph of SHCF gel-forming performance test; (**b**) storage modulus (G′) and loss modulus (G′′) of SHCF gel.

In addition, the results for the elastic modulus of the SHCF show a high elastic modulus of 110.9 ± 9.9 MPa, a tensile strength of 2.8 ± 0.1 MPa, and an elongation of 10.9% ± 1.5 (Table S2). SHCF exhibits excellent mechanical properties, indicating that it can help to close the wound and avoid blood outflow due to the blood pressure of the wound. The gel-forming ability of the SHCF was evaluated (Figure 2a), and it was found that SHCF transformed from a foam to a gel within 5 min of contact with blood. This is because the SHCF is internally connected by non-covalent interactions, allowing it to easily transform into a gel after contact with blood. The storage modulus (G′) of the SHCF gel was 18.37 kPa, demonstrating a high mechanical strength (Figure 2b), indicating better adaptation and adhesion to the wound, which promotes hemostasis.

### 3.3. In Vitro Coagulation and RBC Enrichment Capacity of SHCF

**Figure 3:**
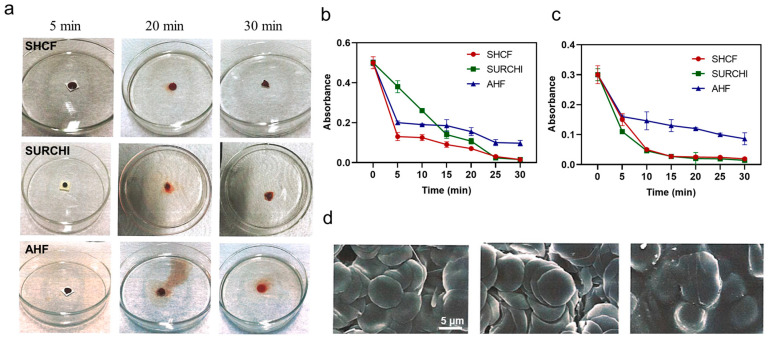
(**a**) Photographs of SHCF, SURCHI, and AHF in contact with blood at different time points in the whole-blood coagulation test; (**b**) absorbance curves of the solutions at different time points in the whole-blood coagulation test and (**c**) RBC adsorption test; (**d**) SEM photographs of the solutions in the RBC adsorption test. Error bars indicate SD (n = 3).

The hemostatic performance of the material was evaluated using a whole-blood coagulation test. The SHCF could effectively coagulate blood and form a stable blood clot within 20 min (Figure 3a). No hemolysis occurred after adding deionized water. In contrast, the control material failed to completely coagulate blood within the same time period, and caused hemolysis. The change in the absorbance of the solution indicated that the SHCF had the lowest absorbance and fastest decrease rates among the three materials at the same time point, all of which were superior to SURCHI and AHF (Figure 3b). Therefore, SHCF can significantly accelerate blood coagulation in vitro and shows a better coagulation speed and effect compared to the two commercial absorbable hemostatic materials.

Moreover, SHCF was able to enrich red blood cells, with a high adsorption rate of up to 50% (Figure 3c,d). The adsorption rate of red blood cells gradually increased with time, which may be due to the porous structure inside the material providing a place for cell adhesion, thereby helping to adsorb more red blood cells and promote the formation of an initial clot.

### 3.4. In Vivo Hemostatic Properties of SHCF

**Figure 4:**
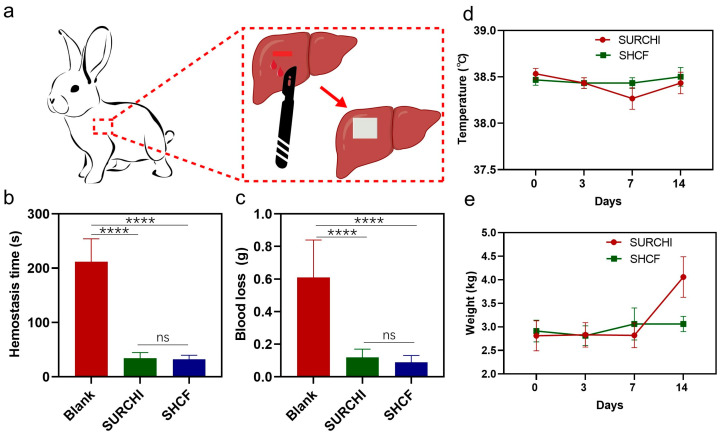
(**a**) Schematic diagram of the rabbit liver injury model; (**b**) hemostatic time and (**c**) blood loss in the blank, SURCHI, and SHCF groups; (**d**) body temperature and (**e**) weight monitoring results in rabbits from 0 to 14 days. Error bars indicate SD (n = 15). **** *p* < 0.0001.

A New Zealand rabbit liver injury model was used to evaluate the in vivo hemostatic performance of the SHCF, with SURCHI used as a control (Figure 4a). The results showed that the average hemostasis times for the SURCHI group and the SHCF group were 34.0 s and 32.1 s, respectively, 84% and 85% shorter than 211.7 s in the blank group (Figure 4b). The SHCF had a 65% reduction in hemostasis time compared to the reported chitosan/gelatin composite foam [8] and a 37% reduction compared to the reported chitosan/gelatin/sodium hyaluronate hemostatic dressing [48], showing a rapid hemostatic effect. The blood loss for the SURCHI group and the SHCF group was 0.19 g and 0.12 g, respectively, which was reduced by 69% and 80% compared to 0.61 g in the blank group (Figure 4c). The SHCF had a short hemostasis time and low blood loss in the rabbit liver bleeding model and a good hemostatic effect on wounds in small animals. In addition, we tested the body temperature and weight of the animals on different days after surgery, and no abnormalities were found (Figure 4d,e). This indicates that the experimental animals recovered well after surgery and that the SHCF did not have adverse effects on the rabbits after hemostasis.

**Figure 5:**
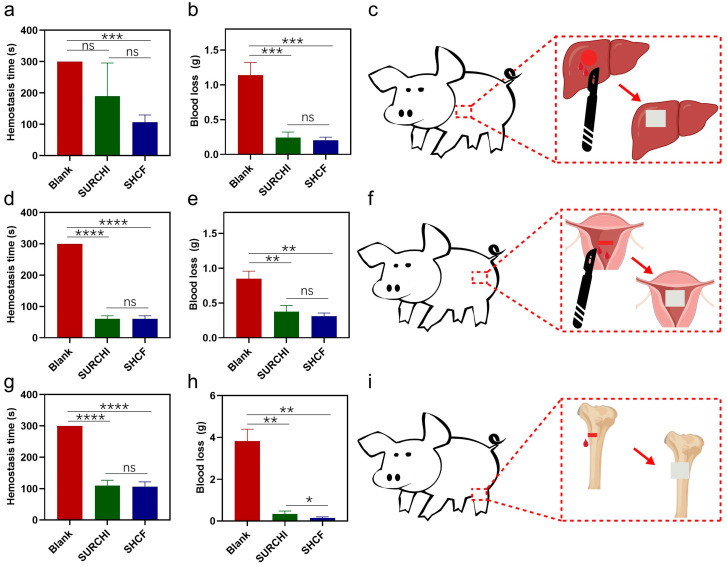
(**a**) Hemostatic time and (**b**) blood loss of the blank, SURCHI, and SHCF groups in the porcine liver injury model; (**c**) schematic diagram of porcine liver injury model; (**d**) hemostatic time and (**e**) blood loss in the blank, SURCHI, and SHCF groups in the porcine uterine wall injury model; (**f**) schematic diagram of porcine uterine wall injury model; (**g**) hemostatic time and (**h**) blood loss in the blank, SURCHI, and SHCF groups in the porcine bone injury model; (**i**) schematic diagram of the porcine bone injury model. Error bar indicates SD (n = 3). * *p* < 0.05, ** *p* < 0.01, *** *p* < 0.001, **** *p* < 0.0001.

In order to further explore the hemostatic effect of the SHCF on severe bleeding and complex wounds, its hemostatic performance was further evaluated using a small porcine injury bleeding model, with SURCHI used as a control. The average hemostasis time in the SHCF group for the porcine liver injury bleeding model was 106.7 s, which was 64% shorter than that of the blank group (300.0 s) and 44% shorter than that of the SURCHI group (190.0 s) (Figure 5a). At the same time, the amount of bleeding in the SHCF group was 0.205 g, an 82% reduction compared to the blank group (1.138 g) (Figure 5b,c). The SHCF had a shorter hemostasis time and less bleeding. For the porcine uterine wall injury bleeding model, the hemostasis time in the SHCF group was 60.0 s, which was 80% shorter than that of the blank group (300.0 s) and comparable to that of the SURCHI group (Figure 5d). The amount of bleeding in the SHCF group was 0.310 g, a 63% reduction compared to the blank group (0.848 g), a 17% reduction compared to the absorbable gauze group (0.374 g), and a greater level than that in the SURCHI group (Figure 5e,f). For the porcine bone injury bleeding model, the hemostasis time in the SHCF group was 107.0 s, which was 64% shorter than that of the blank group (300.0 s) and equivalent to that of the SURCHI group (107.0 s) (Figure 5g). The amount of bleeding in the SHCF group was 0.144 g, which was a significant reduction of 96% compared to the blank group (3.822 g) and a 57% reduction compared to the SURCHI group (0.337 g) (Figure 5h,i).

Compared with rabbits, porcines have greater bleeding and higher difficulty in hemostasis. Therefore, using the porcine injury model to evaluate the hemostatic performance of the hemostatic foam can better simulate its hemostatic effect when applied to human trauma. The SHCF exhibited excellent hemostatic effects in the porcine liver injury, uterine wall injury, and bone injury bleeding models. SHCFs can achieve hemostasis via the following mechanisms. (1) First, they can quickly absorb water from the blood, rapidly enrich blood cells, and accelerate the formation of the initial blood clot at the wound site. Second, they can transform absorbed blood into a gel, thereby filling and sealing the wound, with the gel-like form of SHCFs enabling them to adapt to irregular and complex wounds such as the hemostasis of uterine wall and bone injuries. Third, as SHCFs are richly stimulated by coagulation, they accelerate the generation of fibrin, meaning that under the synergistic effect of the three coagulation stimulations SHCF promotes the formation of a stable blood clot at the wound site. Thus, SHCFs are able to achieve rapid hemostasis for severe bleeding and complex wounds.

### 3.5. Anti-Adhesive Properties of SHCF

**Figure 6:**
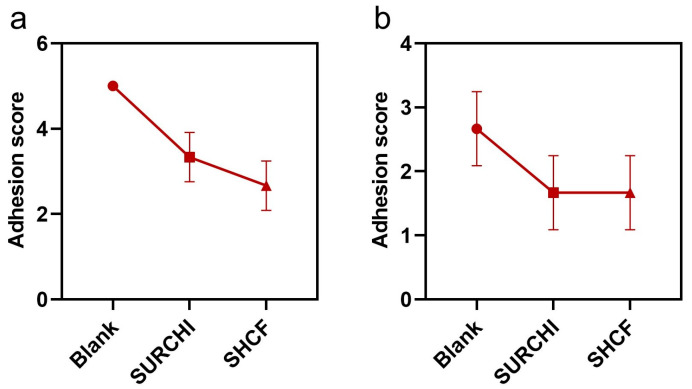
Mean adhesion scores for blank, SURCHI, and SHCF groups in a (**a**) the porcine liver injury model and (**b**) the porcine uterine wall injury model. Error bar indicates SD (n = 3).

Postoperative adhesions can affect organ function and cause serious complications. Therefore, the anti-adhesion properties of SHCF were studied in a porcine liver injury model and uterine wall injury model, using SURCHI as a control. For the porcine liver injury model (Figure 6a), the average adhesion scores of the blank group, SURCHI group, and SHCF group were 5.0, 3.3, and 2.7, respectively. The blank group and SURCHI group had dense adhesions, while the SHCF group had only a small amount of thin foam-like adhesion, which had little effect on the tissue and effectively prevented wound adhesions (** *p* = 0.0075 and ** *p* = 0.0022 correspond to the SURCHI and SHCF groups, respectively). For the uterine wall injury model (Figure 6b), the average adhesion score of the blank group was 2.7, while the average adhesion scores of the SURCHI group and the SHCF group were both low at 1.7 (ns, *p* = 0.1012).

The SHCF effectively reduced the in vivo adhesion score and prevented tissue adhesion. SHCFs can have an anti-adhesion effect through several mechanisms. First, they can act as a barrier to prevent adhesion; high-molecular-weight sodium hyaluronate has good barrier properties due to its large size, meaning that it can occupy more space and reduce friction between tissues and organs, thereby reducing adhesion. Second, they inhibit the proliferation and migration of fibroblasts, thereby preventing adhesion. The inherent negative charge of hyaluronic acid has inhibitory effects on the proliferation and migration of fibroblasts [49,50], while chitosan can effectively inhibit the proliferation and migration of fibroblasts [4,51]. A study by Chen et al. showed that hydrogels containing hyaluronic acid and chitosan had the highest reduction of fibroblast migration in vitro [51]. Therefore, SHCFs obtained from a composite of sodium hyaluronate and carboxymethyl chitosan should have a good inhibitory effect on fibroblast migration. Third, they prevent adhesion by avoiding wound infection and hematoma. The SHCF synthesized in this research showed excellent biocompatibility and safety and did not cause inflammatory reactions in vivo, effectively avoiding adhesion caused by wound inflammation or disease.

### 3.6. Biosafety of SHCF

**Figure 7:**
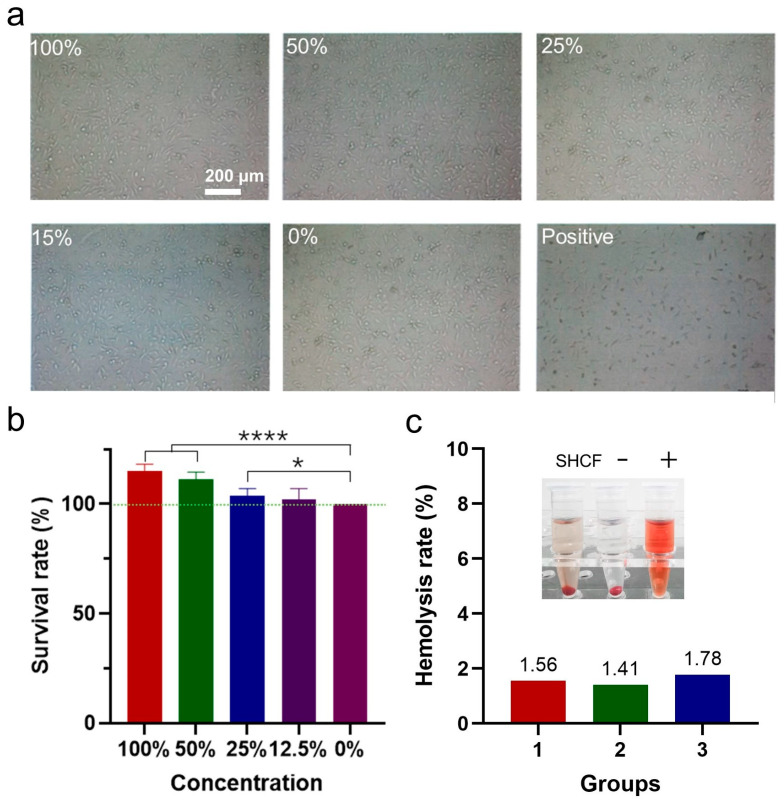
(**a**) Microscopic photographs of cells from 100%, 50%, 25%, 15%, and 0% sample concentration groups and positive control groups; (**b**) cell viability of different groups; (**c**) hemolysis rate of SHCF (3 mg/mL). (−) represents negative control group, (+) represents positive control group. Error bar indicates SD (n = 3). * *p* < 0.05, **** *p* < 0.0001.

The biocompatibility of the SHCF was evaluated via measurement of its cytotoxicity and hemolysis rate [52]. SEM showed that the cells were in good condition without obvious death under different ratios of SHCF addition (Figure 7a). The survival rates of cells in all groups of SHCF were above 100%, demonstrating excellent cell compatibility (Figure 7b). In addition, the hemolysis rate of SHCF was less than 2%, indicating no dissolution effect on red blood cells and good blood compatibility (Figure 7c). The SHCF did not trigger an increase in the levels of the proinflammatory cytokines IL-1β, IL-6, or TNF-α (Figures S2–S4). Therefore, the SHCF has excellent biocompatibility.

### 3.7. Degradation and Metabolic Properties of SHCF

**Figure 8:**
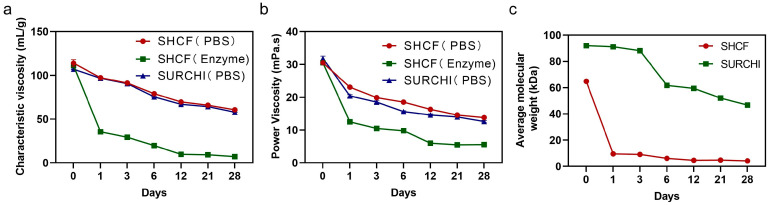
(**a**) Characteristic viscosities of SHCF and SURCHI for different numbers of days in PBS and enzyme solutions; (**b**) kinetic viscosities of SHCF and SURCHI for different numbers of days in PBS and enzyme solutions; (**c**) average molecular weights of SHCF and SURCHI for different numbers of days of degradation. Error bar indicates SD (n = 3).

In order to study the degradation of the SHCF, the degradation of hemostatic materials in the body was simulated in vitro using PBS and enzyme solution. In PBS solution, the intrinsic viscosity of the SHCF continued to decrease from 114.11 mL/g to 60.73 mL/g within 28 days (Figure 8a). In the enzyme solution, the intrinsic viscosity of the SHCF significantly decreased from 111.33 mL/g to 35.53 mL/g on the first day. The dynamic viscosity of the SHCF decreased from 30.5 mPa.s to 5.57 mPa.s in the enzyme solution, and the decrease rate was significantly faster than in the PBS solution (Figure 8b). This indicates that the absorbable hemostatic foam has a greater decrease in dynamic viscosity and can decrease over a shorter period of time in the enzyme solution. The results of the average molecular weight measurement during the SHCF degradation process show that the molecular weight of the SHCF decreased by 93.52% from 64.82 kDa to 4.198 kDa on the first day (Figure 8c). In comparison, SURCHI only degraded by 1% on the first day, and the average molecular weight decreased from 91.98 kDa to 46.73 kDa within 28 days; this is only a 49% reduction, which is much slower than that of the SHCF. Therefore, the SHCF had rapid degradation ability in vitro that is superior to commercially available absorbable hemostatic materials.

**Figure 9:**
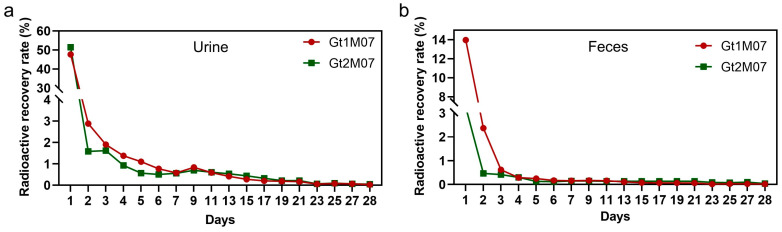
Radioactivity recovery rates of Gt1M07 and Gt2M07 in (**a**) urine and (**b**) feces of rats for different numbers of days. Error bar indicates SD (n = 7).

Metabolism studies in vivo have shown that the main metabolites of SHCFs are excreted through urine, accounting for about 60% of the administered dose (Figure 9a), while another part is eliminated through feces, accounting for about 25% of the administered dose (Figure 9b). Within the first two days, the radioactivity recovery rates in urine and feces sharply decreased from 50% and 14% to around 1.5% and 0.5%, respectively. This indicates that the SHCF can be rapidly degraded and metabolized out of the body, and is mostly metabolized within 23 days. The SHCF was completely degraded in about 3 weeks, and the degradation cycle was shorter than the 6 weeks reported in the literature for a chitosan/gelatin composite absorbable hemostatic sponge [49]. The SHCF with non-covalent bonding is more readily degraded than covalently attached hemostatic materials, exhibiting rapid degradation and metabolism capabilities.

### 3.8. Absorbable Properties of SHCF

**Figure 10:**
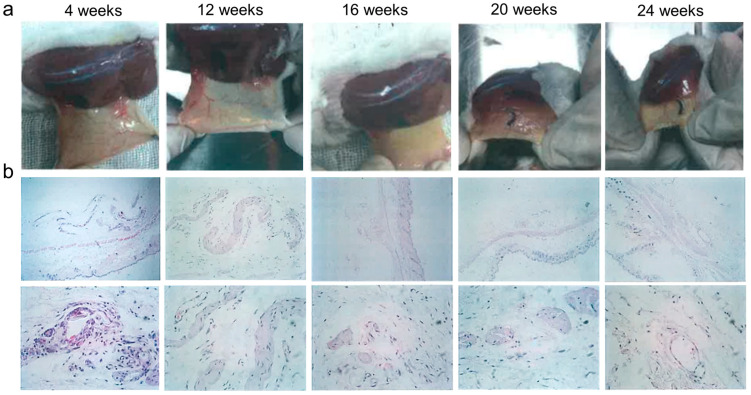
(**a**) Photographs and (**b**) HE-stained images of implantation sites in mice at 4, 12, 16, 20, and 24 weeks.

To further verify the absorbability of the SHCF, its in vivo degradation and metabolism were investigated via subcutaneous implantation experiments in mice. It was found that after 4 weeks no material residue was observed at the implantation site and that the wound had completely healed (Figure 10a). As shown in Figure 10b and Table 1, inflammatory response, angiogenesis, material degradation, and new tissue formation were evaluated by quantitative analysis [53]. An increase in the inflammatory response was confirmed at week 4 and found to decrease with increasing implantation time. The analysis of tissue sections revealed that the material was gradually degraded and absorbed with increasing implantation time, forming new tissue. In addition, minimal (starting at week 12) to moderate (week 16) capillary proliferation was observed. All these results show that SHCF can be spontaneously degraded in vivo without causing significant inflammatory reactions and without affecting wound healing while maintaining good degradability and safety.

## 4. Conclusions

We developed a multi-functional hemostatic foam with rapid hemostasis, anti-adhesion, and rapid degradation properties. SHCFs have rapid fluid absorption, hematocrit enrichment, gel-forming, and coagulation stimulation properties. The hemostatic performance of the synthesized SHCF was verified using a rabbit liver hemorrhage model, showing an 85% reduction in hemostatic time and an 80% reduction in blood loss. More importantly, due to its gel-forming properties and multiple coagulation effects the SHCF was able to effectively control bleeding in three severe and complex porcine hemorrhage models, namely, liver injury, uterine wall injury, and bone injury. The synthesized SHCF effectively prevented tissue adhesion, completely degraded in a short time, and was almost completely metabolized within three weeks. These results show that the absorbable hemostatic foam SHCF effectively controlled bleeding in complex wounds via the combined action of multiple hemostatic mechanisms, prevented post-operative wound adhesions, and was degraded and metabolized out of the body. Therefore, the SHCF synthesized in this research represents a multifunctional absorbable hemostatic foam with great potential in wound-healing applications.

## Figures and Tables

**Table 1:** Evaluation of the H&E morphological characteristics following tissue implantation of SHCF.

	H&E Morphological Characteristics of Tissue			
	Inflammatory Response	Angiogenesis	Material Degradation	New Tissue Formation
Week 4	+++	0	+	+/++
Week 12	+/++	++	+++	+/++
Week 16	+/++	+++	+++	+/++
Week 20	+	0	+++	++
Week 24	+	0	+++	++

Note: Scores based on a semi-quantitative scale in which 0 reflects absence, + low level, ++ medium level, and +++ high level for the accessed parameter.

## Data Availability

Not applicable.

## Supplementary Materials

The following supporting information can be downloaded at: https://www.mdpi.com/article/10.3390/bioengineering10070868/s1, Table S1: BET analysis of SHCF; Table S2: Elastic modulus of SHCF; Table S3: Contact angle and surface free energy of SHCF; Figure S1: Contact angle picture of water droplets with SHCF; Figure S2: IL-1β concentration values in the blank and SHCF groups; Figure S3: IL-6 concentration values in the blank and SHCF groups; Figure S4: TNF-α concentration values in the blank and SHCF groups.
