# Supplementary material for: Multifunctional Sodium Hyaluronate/Chitosan Foam Used as an Absorbable Hemostatic Material
Source: Bioengineering (Basel). 2023 Jul 21;10(7):868. doi: 10.3390/bioengineering10070868 (PMC10376295; doi:10.3390/bioengineering10070868)
Supplement: Supplementary file 1 [file bioengineering-10-00868-s001.zip › bioengineering-2449765-supplementary.pdf]

Supplementary Materials

Table S1 BET analysis of SHCF

Sample	Surface area (m^2/g)	Pore volume (cm^3/g)
SHCF	6.974	0.007

Table S2. Elastic modulus of SHCF

Groups	1	2	3	Mean, SD
Elastic modulus (MPa)	98.8	123.1	110.9	110.9±9.9
Tensile strength (MPa)	2.7	3.0	2.8	2.8±0.1
Elongation (%)	12.0	12.0	8.8	10.9±1.5

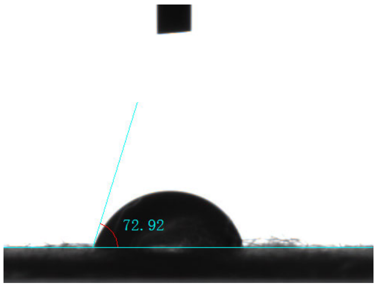

Figure S1. Contact angle picture of water droplets with SHCF

Table S3. Contact angle and surface free energy of SHCF

Sample	Contact angle θ (°)	Surface free energy ($mJ \cdot m^{-2}$)
SHCF	72.01 ± 0.91	37.30 ± 1.4

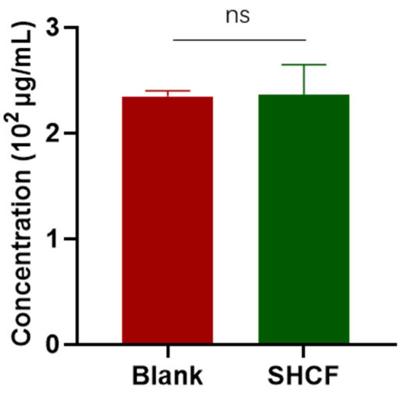

Figure S2. IL-1β concentration values in the blank and SHCF groups. Error bar indicate S.D (n = 3), $^{ns}p > 0.05$.

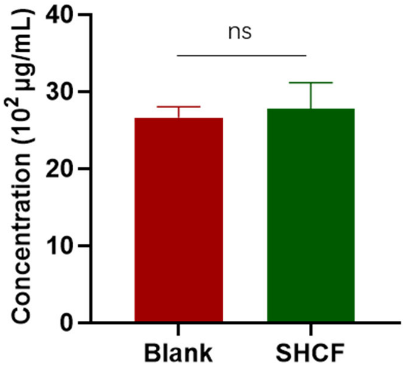

Figure S3. IL-6 concentration values in the blank and SHCF groups. Error bar indicate S.D (n = 3), $^{ns}p > 0.05$.

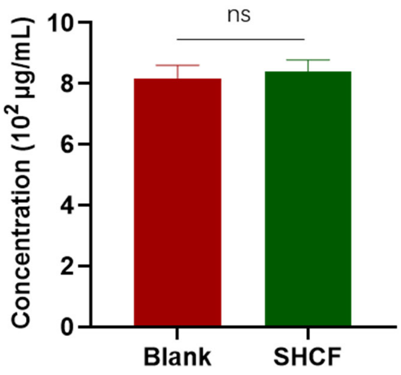

Figure S4. TNF-α concentration values in the blank and SHCF groups. Error bar indicate S.D (n = 3), $^{ns}p > 0.05$.
